# Bacteriological Diagnosis: Tabular Aids for Use in Practical Work

**Published:** 1892-09

**Authors:** 


*Bacteriological Diagnosis: Tabular Aids for use in Practical Work.* By JAMES EISENBERG, Ph.D., M.D. Translated by NORVAL H. PIERCE, M.D. Pp. xiv., 184. Philadelphia: THE F. A. DAVIS CO. 1892.

The object of this book is to provide concise tables of information for those who study bacteria systematically; each page is devoted to a short account of the methods of growth, characteristics, and *habitat* of a single microbe. The classification adopted is into non-pathogenic and pathogenic bacteria, and the former are sub-divided into those that liquefy and those that do not liquefy gelatine. There is a useful appendix on the *technique* of staining and cultivating. The book has gone through the test of a second edition in German, and shows evidence of real practical knowledge.

Experiments have not apparently been confined to the lower animals, for we read that the streptococcus erysipelatis has been "successfully inoculated from person to person"; also that "a pure culture of the 'gonococcus' inoculated on the urethra of a woman produced a severe gonorrhœa."

The translator has not always been very happy in his rendering into English, or rather into American. He says that the comma bacillus has been found in "recently moribund cholera patients"; the typhoid bacillus is called the "bacillus typhosus"; instead of using the Greek "$\mu$" to indicate micro-millimetres, he writes m. or mm., and states (for example) that the sarcina ventriculi "is 25 m. in diameter," which would make it the size of a large house. He uses the word bacteri*æ* as the plural of bacteri*a*, and elsewhere gets over the difficulty of termination by calling them *bacteries*. It is always easy, however, to follow the meaning, and the bulk of the work is carefully translated. The book ought to be very useful to students of the rapidly extending science of bacteriology.

